# Peripheral Blood T Cell Dynamics Predict Relapse in Multiple Sclerosis Patients on Fingolimod

**DOI:** 10.1371/journal.pone.0124923

**Published:** 2015-04-28

**Authors:** Zi-Ye Song, Ryo Yamasaki, Yuji Kawano, Shinya Sato, Katsuhisa Masaki, Satoshi Yoshimura, Dai Matsuse, Hiroyuki Murai, Takuya Matsushita, Jun-ichi Kira

**Affiliations:** 1 Department of Neurology, Neurological Institute, Graduate School of Medical Sciences, Kyushu University, Fukuoka, Japan; 2 Department of Neurological Therapeutics, Neurological Institute, Graduate School of Medical Sciences, Kyushu University, Fukuoka, Japan; Research Inst. of Environmental Med., Nagoya Univ., JAPAN

## Abstract

**Background:**

Fingolimod efficiently reduces multiple sclerosis (MS) relapse by inhibiting lymphocyte egress from lymph nodes through down-modulation of sphingosine 1-phosphate (S1P) receptors. We aimed to clarify the alterations in peripheral blood T cell subsets associated with MS relapse on fingolimod.

**Methods/Principal Findings:**

Blood samples successively collected from 23 relapsing-remitting MS patients before and during fingolimod therapy (0.5 mg/day) for 12 months and 18 healthy controls (HCs) were analysed for T cell subsets by flow cytometry. In MS patients, the percentages of central memory T (CCR7^+^CD45RO^+^) cells (TCM) and naïve T (CCR7^+^CD45RO^-^) cells decreased significantly, while those of effector memory T (CCR7^-^CD45RA^-^) and suppressor precursor T (CD28^-^) cells increased in both CD4^+^T and CD8^+^T cells from 2 weeks to 12 months during fingolimod therapy. The percentages of regulatory T (CD4^+^CD25^high^CD127^low^) cells in CD4^+^T cells and CCR7^-^CD45RA^+^T cells in CD8^+^T cells also increased significantly. Eight relapsed patients demonstrated greater percentages of CD4^+^TCM than 15 non-relapsed patients at 3 and 6 months (*p*=0.0051 and *p*=0.0088, respectively). The IL17-, IL9-, and IL4-producing CD4^+^T cell percentages were significantly higher at pre-treatment in MS patients compared with HCs (*p*<0.01 for all), while the IL17-producing CD4^+^T cell percentages tended to show a transient increase at 2 weeks of fingolimod therapy (*p^corr^*=0.0834).

**Conclusions:**

The CD4^+^TCM percentages at 2 weeks to 12 months during fingolimod therapy are related to relapse.

## Introduction

Multiple sclerosis (MS) is an inflammatory demyelinating disease of the central nervous system and is assumed to be an autoimmune disease targeting myelin [1]. Fingolimod efficiently reduces MS relapse by inhibiting lymphocyte egress from lymph nodes through down-modulation of sphingosine 1-phosphate (S1P) receptors [2,3], which is consistent with findings in an animal model of MS that fingolimod successfully reduced the infiltration of myelin antigen-specific CD4^+^T cells into inflammatory sites in experimental autoimmune encephalomyelitis [4]. Fingolimod traps CD45RO^+^ central memory T cells (TCM) and naïve T cells expressing homing receptors for lymph nodes, CCR7 and CD62L, within lymph nodes [5]. The drug is supposed to exert its effects by inhibiting recirculation of TCM, which include autoreactive T cells [6]. Although fingolimod was also found to be beneficial in a controlled trial for human renal transplantation [7], its effects have not been examined for any other autoimmune diseases in large-scale clinical trials.

MS is now assumed to be a T helper 1 (Th1)/Th17-mediated autoimmune disease [1]. Some authors reported a decrease in IL17-positive cells in MS patients on fingolimod 1.25 mg once daily at the long-term therapy stage, but not on IFNβ [8], while others reported an increase in IL17-producing cells in a considerable fraction of MS patients on fingolimod 0.5 mg once daily at the short-term treatment stage [9]. In addition, effector memory T cells (TEM) without CCR7, which reside in non-lymphoid organs and mainly comprise IFNγ-producing Th1 cells protecting against infection, were found to show a relative increase during fingolimod therapy [7], while other authors reported TEM as the principal IL17-producing T cells [10]. Thus, the short-term and long-term effects of fingolimod on Th17 cells at an ordinary dosage (0.5 mg/day) remain to be elucidated. Therefore, we aimed to clarify the alterations in peripheral blood T cell subsets during short-term and long-term treatment periods, which are associated with therapeutic efficacy or treatment failure including suboptimal responses during fingolimod therapy.

## Materials and Methods

### Patients and control subjects

Venous blood samples were obtained from 18 healthy controls (HCs) (12 females and 6 males; mean age ± SD, 41.2±12.2 years) and 23 relapsing-remitting MS patients (15 females and 8 males; mean age ± SD, 42.3±12.5 years) who were started on fingolimod therapy between 2012 and 2014 at Kyushu University Hospital after informed consent was obtained (Table 1). In the latter, blood was withdrawn before and at 2 weeks and 1, 2, 3, 6, and 12 months after initiation of fingolimod 0.5 mg once daily. MS was diagnosed based on the 2005 revised McDonald criteria for MS [11] and all patients were seronegative for anti-aquaporin-4 (AQP4) antibodies [12,13]. Ten patients had neither immunomodulatory drugs nor corticosteroids within 3 months before the initiation of fingolimod, two had IFNβ-1b until 10 days and 1 day before fingolimod initiation, respectively, one had IFNβ-1a until 14 days before, and nine had prednisolone until 1 day before. All MS patients underwent a neurological examination at our outpatient clinic at 2–4-week intervals and were evaluated by the Expanded Disability Status Scale of Kurtzke (EDSS) [14] with calculation of the Progression Index [15], and observed for the emergence of clinical relapse. Clinical relapse was defined as the appearance of new symptoms or return of old symptoms for a period of 24 hours or more in the absence of a change in the core body temperature or infection [16]. Brain and spinal cord MRI was performed at 6 and 12 months after initiation of fingolimod to determine the presence of new/enlarging T2 lesions and gadolinium-enhanced T1 lesions (MRI relapse) [16]. All MRI studies were performed using a 1.5 T unit (Magnetom Vision and Symphony; Siemens Medical Systems, Erlangen, Germany) as previously described [17]. This study was approved by the Kyushu University Hospital Ethics Committee. All individuals involved in this study signed a written informed consent form (Approval Number: 575–03).

**Table 1:** Demographic features of the subjects.

	HCs	MS PT	MS during fingolimod treatment for 12 months	
			Relapsed patient	Non-relapsed patient
Sex (male: female)	6: 12	8: 15	3: 5	5: 10
Age at examination (mean ± SD, years)	41.2 ± 12.2	42.3 ± 12.5	35.6 ± 13.3	40.0 ± 11.9
Age at Onset (mean ± SD, years)	NA	28.9 ± 12.4	28.1 ± 12.4	28.7 ± 12.8
Disease duration (mean±SD, years)	NA	11.2 ± 9.7	7.3 ± 8.1	13.2 ± 10.1
Annualised relapse rate (mean ± SD, /years)	NA	3.3 ± 3.0	2.9 ± 3.0	3.5 ± 3.1
EDSS scores before fingolimod treatment (mean ± SD)	NA	2.8 ± 2.1	2.5 ± 2.3	3.2 ± 2.1
Progression Index (mean ± SD)	NA	0.6 ± 1.0	0.9 ± 1.6	0.4 ± 0.4

Abbreviations: HCs = healthy controls; MS PT = multiple sclerosis at pre-treatment; EDSS = Expanded Disability Status Scale of Kurtzke.

### Antibodies and flow cytometry

T cells were analysed in whole blood specimens using the following antibodies from Miltenyi Biotec (Auburn, CA): anti-human CD3-VioBlue (BW264/56, MACS), CD4-APC (M-T466, MACS), CD8-APC (BW135/80, MACS), CD45RO-FITC (UCHL1, MACS), CD45RA-APC-Vio770 (T6D11, MACS), CD127-FITC (MB15-18C9, MACS), CD25-PE (4E3, MACS), CCR7-PE (FR11-11E8, MACS), CD8-PE (BW135/80, MACS), CD4-FITC (M-T466, MACS), and CD28-APC (15E8, MACS). The following isotype controls from Miltenyi Biotec were also used: anti-mouse IgG1-PE (IS5-21F5, MACS), IgG1-APC (IS5-21F5, MACS), IgG1-FITC (IS5-21F5, MACS), IgG2a-APC (S43.10, MACS), IgG2a-VioBlue (S43.10, MACS), IgG2a-PE (S43.10, MACS), IgG2a-FITC (S43.10, MACS), IgG2b-APC-Vio770 (IS6-11E5.11, MACS), IgG2b-PE (IS6-11E5.11, MACS), and IgG2b-FITC (IS6-11E5.11, MACS). Specific cytokine-producing cells and chemokine receptor-positive cells were analysed using the following antibodies: anti-human CD4-APC (M-T466, MACS; Miltenyi Biotec), CD8-VioBlue (BW135/80, MACS; Miltenyi Biotec), IFNγ-FITC (25723.11; BD Biosciences, San Jose, CA), IL17-PE (BL168; Biolegend, San Diego, CA), IL4-FITC (MP4-25D2; Biolegend), and IL9-PE (AH9R7; Biolegend). Peripheral blood mononuclear cells (PBMCs) were isolated with Lymphoprep Tubes (AXIS-SHIELD Poc AS, Oslo, Norway). For intracellular cytokine detection, PBMCs were stimulated with phorbol 12-myristate 13-acetate (10 ng/ml) and ionomycin (1 μg/ml) (both from Enzo Life Sciences, Plymouth Meeting, PA) in the presence of brefeldin A (Sigma-Aldrich, St. Louis, MO) for 4 h at 37°C in 5% CO_2_, fixed, and permeabilised with BD FACS lysing solution and BD FACS permeabilising solution (BD Biosciences) according to the manufacturer’s instructions. Data were acquired using a FACScan flow cytometer (MACSQuant Analyzer; Miltenyi Biotec) according to standard procedures for whole blood samples and PBMCs, as described previously [18,19]. Naïve T (CCR7^+^CD45RO^-^) cells, TCM (CCR7^+^CD45RO^+^), TEM (CCR7^-^CD45RA^-^), CD8^+^CD45RA^+^effector memory T (CD8^+^ CCR7^-^CD45RA^+^) cells (TEMRA), regulatory T (CD4^+^CD25^high^CD127^low^) cells (Treg), and suppressor precursor T (CD28^-^) cells (Ts) were measured in all 23 patients in S1 Fig, while IFNγ-, IL17-, IL9-, and IL4-producing cells were analysed in 16 patients.

### Statistical analysis

Demographic features between MS patients and HCs and between MS patients with and without relapse on fingolimod were compared by the Mann—Whitney U test, except for the sex ratio compared by Fisher’s test. T cell counts and subset percentages between MS patients and HCs, between pre-treatment and post-treatment values in MS patients, and between MS patients with and without relapse on fingolimod were compared by the Mann—Whitney U test. For independent multiple comparisons, uncorrected *p* (*p* ^*uncorr*^) values were multiplied by the number of comparisons to calculate corrected *p* (*p* ^*corr*^) values (Bonferroni—Dunn’s correction). Values of *p*<0.05 were considered to indicate statistical significance.

## Results

### Demographic features and treatment response to fingolimod

During fingolimod therapy, four patients had mild liver dysfunction and three patients had lymphopenia (less than 200 /μl), all of which recovered after cessation of the drug or symptomatic treatment. Also during fingolimod therapy, two patients had clinical relapses (both at 3 months after initiation of the drug) and six patients had new gadolinium-enhanced lesions on T1-weighted MRI or new/enlarging lesions on T2-weighted images at 6 or 12 months after initiation of the drug. These eight patients were classified as a relapsed group, while the remaining 15 patients were classified as a non-relapsed group. Although the relapsed patients had a shorter disease duration and higher Progression Index than the non-relapsed patients, there were no significant differences in any of the demographic features examined between the relapsed and non-relapsed patients (Table 1).

### Comparisons of T cell subsets between MS patients at pre-treatment and HCs

**Fig 1:**
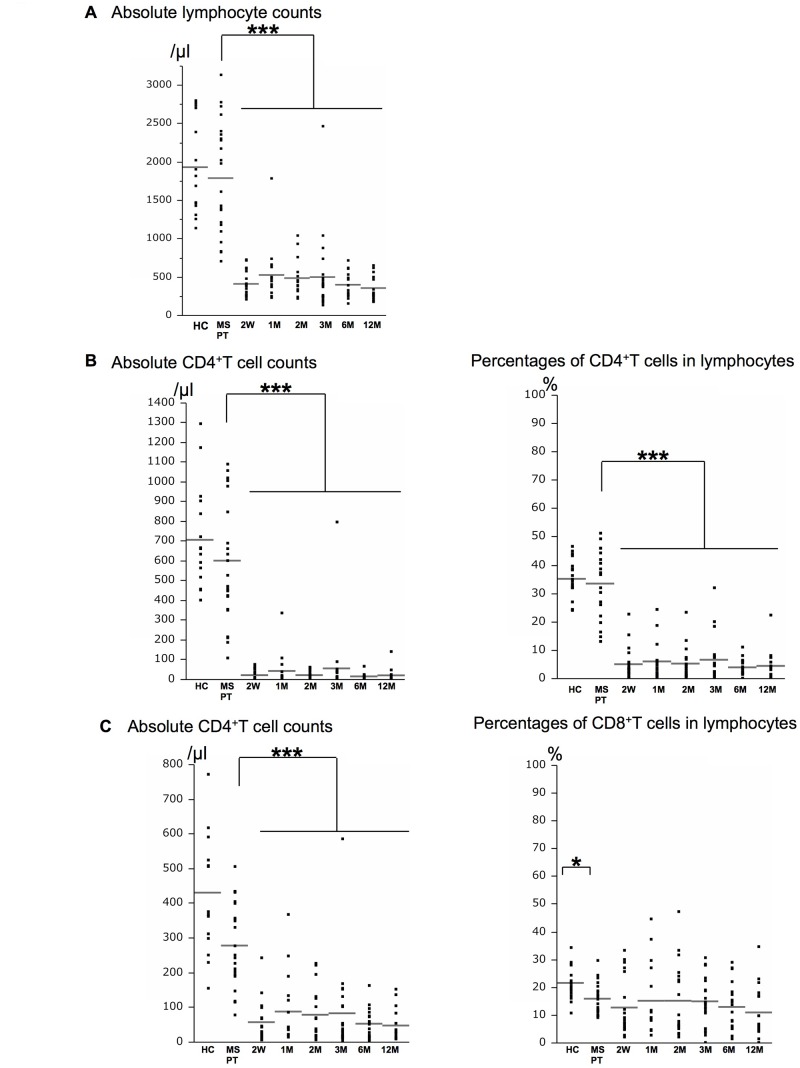
Counts of lymphocytes, CD4^+^T, and CD8^+^T cells are decreased from 2 weeks. Effects of fingolimod on peripheral blood lymphocyte counts (A) and absolute numbers and percentages of CD4^**+**^T (B) and CD8^**+**^T (C) cells in lymphocytes of healthy controls (HCs) and MS patients at pre-treatment (MS PT) and the indicated periods of fingolimod treatment. The numbers examined at each time point were: HC = 18, and MS PT = 23, 2W = 20, 1M = 17, 2M = 19, 3M = 23, 6M = 20, 12M = 18. The horizontal bars indicate the mean values. W = week; M = month. ***p<0.0001, *p<0.05.

**Fig 2:**
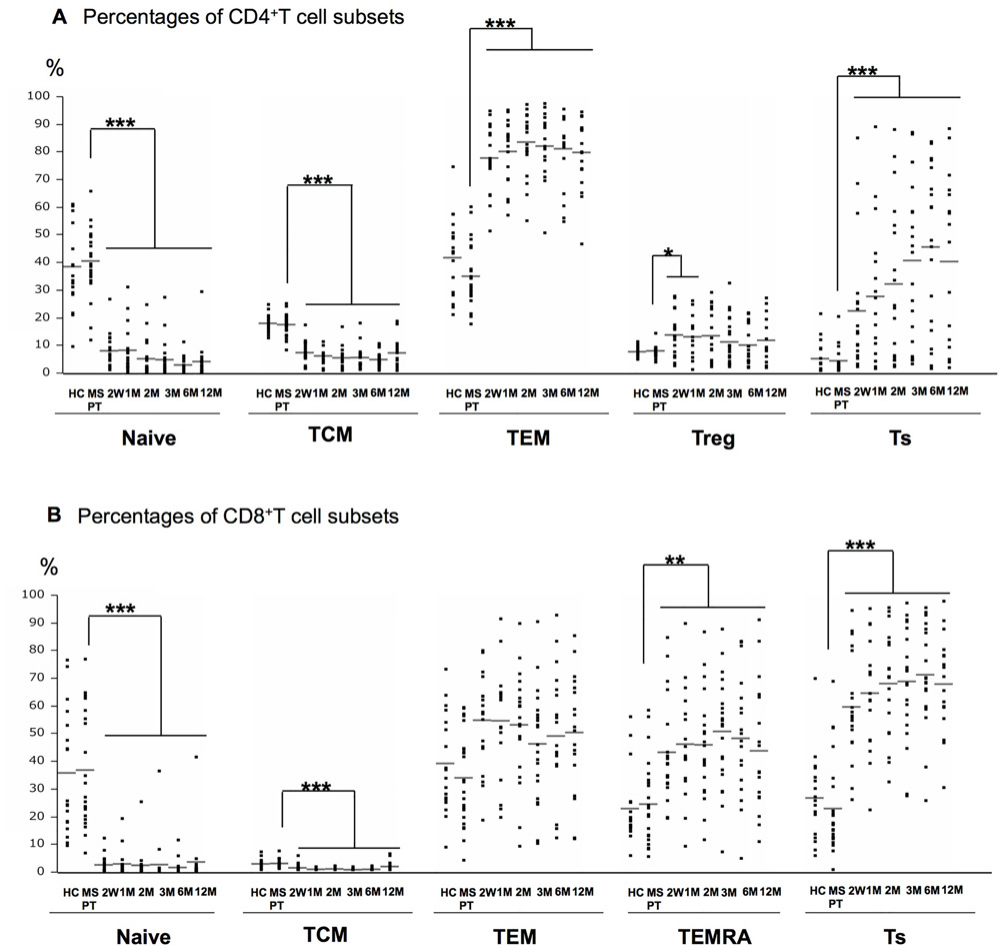
The percentages of T cell subsets show significant changes from 2 weeks. Effects of fingolimod on phenotypically distinct CD4^+^T (A) and CD8^+^T (B) cell subpopulations in healthy controls (HCs) and MS patients at pre-treatment (MS PT) and the indicated periods of fingolimod treatment. Naïve = naïve T cells (CCR7^+^CD45RO^-^); TCM = central memory T cells (CCR7^+^CD45RO^+^); TEM = effector memory T cells (CCR7^-^CD45RA^-^); Treg = regulatory T cells (CD4^+^CD25^high^CD127^low^); Ts = suppressor precursor T cells (CD28^-^); TEMRA = CD8^+^CD45RA^+^effector memory T cells (CD8^+^CCR7^-^CD45RA^+^). The numbers examined at each time point were: HC = 18, and MS PT = 23, 2W = 20, 1M = 17, 2M = 19, 3M = 23, 6M = 20, 12M = 18. The horizontal bars indicate the mean values. W = week; M = month. ***p<0.0001, **p<0.01, *p<0.05.

There were no significant differences in the counts of lymphocytes, CD4^+^T cells, and CD8^+^T cells and the percentages of CD4^+^T cells between the MS patients at pre-treatment and HCs, while the percentage of CD8^+^T cells was significantly lower in MS patients than in HCs (*p*<0.05) (Fig 1A–1C). In addition, there were no significant differences in the percentages of naïve T cells, TCM, TEM, Treg, and Ts in CD4^+^T cells and naïve T cells, TCM, TEM, TEMRA, and Ts in CD8^+^T cells between the MS patients at pre-treatment and HCs (Fig 2A and 2B).

### Alterations in T cell subsets during fingolimod therapy

The total lymphocyte counts were markedly decreased from 2 weeks until 12 months after initiation of fingolimod (Fig 1A), and the absolute counts and percentages of CD4^+^T cells and the counts of CD8^+^T cells were also decreased (Fig 1B and 1C). The absolute counts of TCM, naïve T cells, and TEM in both CD4^+^T and CD8^+^T cells and Treg in CD4^+^T cells decreased significantly from 2 weeks to 12 months compared with the pre-treatment levels (*p*<0.0001 for all), as shown in S2 Fig.

In CD4^+^T cells, the percentages of TCM and naïve T cells decreased significantly from 2 weeks to 12 months compared with the pre-treatment levels (*p*<0.0001 for all), while the TEM and Ts percentages increased from 2 weeks to 12 months compared with the pre-treatment levels (*p*<0.0001 for all) and the Treg percentages also increased from 2 weeks to 1 month (*p*<0.05) (Fig 2A). In CD8^+^T cells, the percentages of naïve T cells and TCM decreased significantly from 2 weeks to 12 months compared with the pre-treatment levels, while the TEMRA and Ts percentages increased from 2 weeks to 12 months (*p*<0.0001 for all) (Fig 2B). However, the TEM percentages in CD8^+^T cells showed no significant changes.

### Comparisons of specific cytokine-producing T cell percentages between MS patients at pre-treatment and HCs

**Fig 3:**
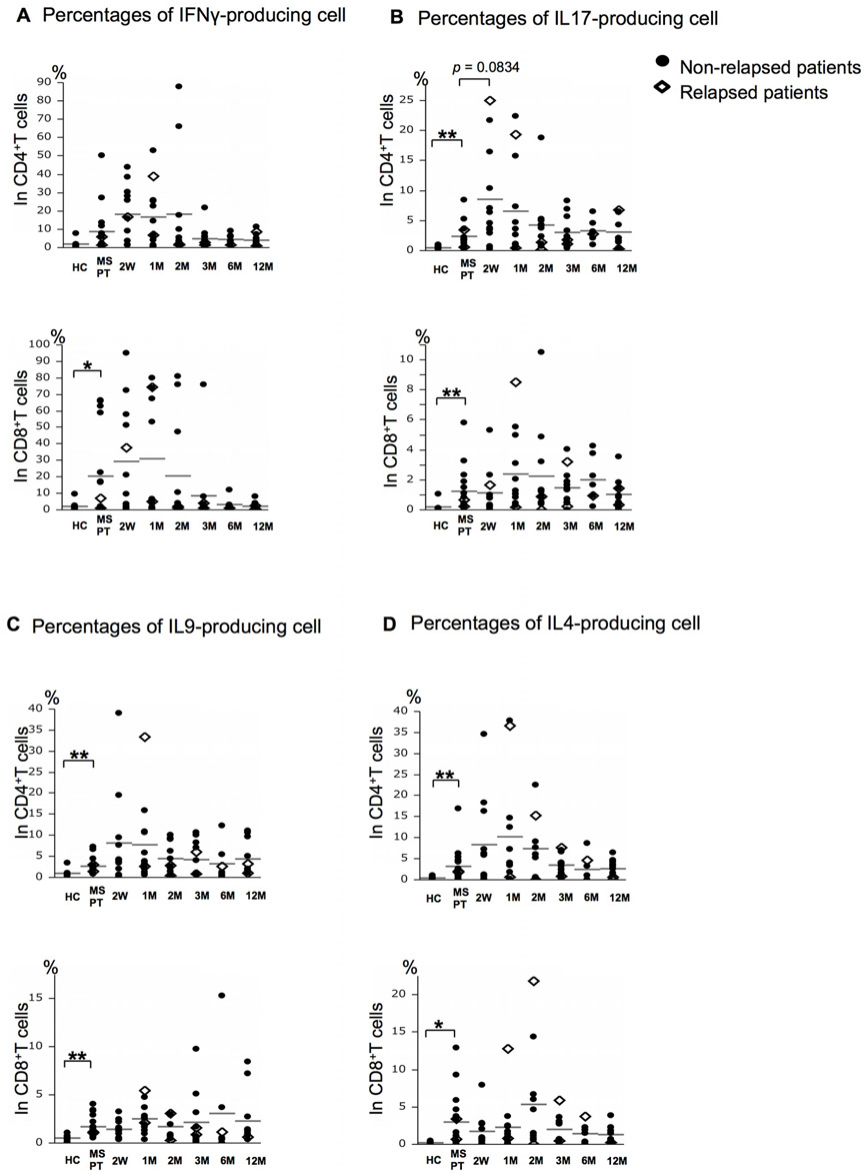
The IL17-, IL9-, and IL4-producing cells are higher in MS patients than in HCs. Effects of fingolimod on IFNγ- (A), IL17- (B), IL9- (C), and IL4-producing (D) cells in CD4^+^T and CD8^+^T cells in healthy controls (HCs) and MS patients at pre-treatment (MS PT) and the indicated periods of fingolimod treatment. The numbers examined at each time point were: HC = 9, and MS PT = 16, 2W = 12, 1M = 12, 2M = 11, 3M = 12, 6M = 7, 12M = 11. The horizontal bars indicate the mean values. Open diamonds = MS patients with relapse during fingolimod therapy (*n* = 2); closed circles = MS patients without relapse during the therapy (*n* = 14). W = week; M = month. **p<0.01, *p<0.05.

The percentages of IL17-, IL9-, and IL4-producing cells in CD4^+^T cells were significantly higher in MS patients than in HCs (*p*<0.01 for all), and the percentages of IFNγ-, IL17-, IL9-, and IL4-producing cells in CD8^+^T cells were also significantly higher in MS patients than in HCs (*p*<0.05, *p*<0.01, *p*<0.01, and *p*<0.05, respectively) (Fig 3). These trends were observed in IL17-, IL9-, and IL4-producing CD4^+^T and CD8^+^T cells in the MS patients, irrespective of whether they had IFNβ or corticosteroids within 3 months of initiation of fingolimod, as shown in S3 Fig. However, the increases in IFNγ-producing CD4^+^T and CD8^+^T cells at pre-treatment were only significant in the MS patients without IFNβ or corticosteroids.

### Alterations in specific cytokine-producing T cell percentages during fingolimod therapy

The percentages of IFNγ-producing cells in both CD4^+^T and CD8^+^T cells and those of IL17-producing cells in CD4^+^T cells increased transiently at 2 weeks after initiation of fingolimod (*p* ^*uncorr*^<0.05 for all, but not significant after correction for multiple comparisons except that IL17-producing CD4^+^T cells showed an increased tendency at 2 weeks, *p* ^*corr*^ = 0.0834), and thereafter decreased gradually to the pre-treatment levels after 3 months (Fig 3). These changes were observed regardless of prior treatment with IFNγ or prednisolone (data not shown). In contrast, IL17-producing CD8^+^T cells, and IL4- and IL9-producing CD4^+^T and CD8^+^T cells showed no such transient increases after the introduction of fingolimod. However, the absolute counts of all cytokine-producing T cells in CD4^+^T cells decreased significantly from 2 weeks to 12 months compared with the pre-treatment levels (*p* ^*corr*^<0.05 for all), as shown in S4 Fig.

### Correlations of alterations in distinct T cell subsets and specific cytokine-producing T cells during fingolimod therapy with treatment response

**Fig 4:**
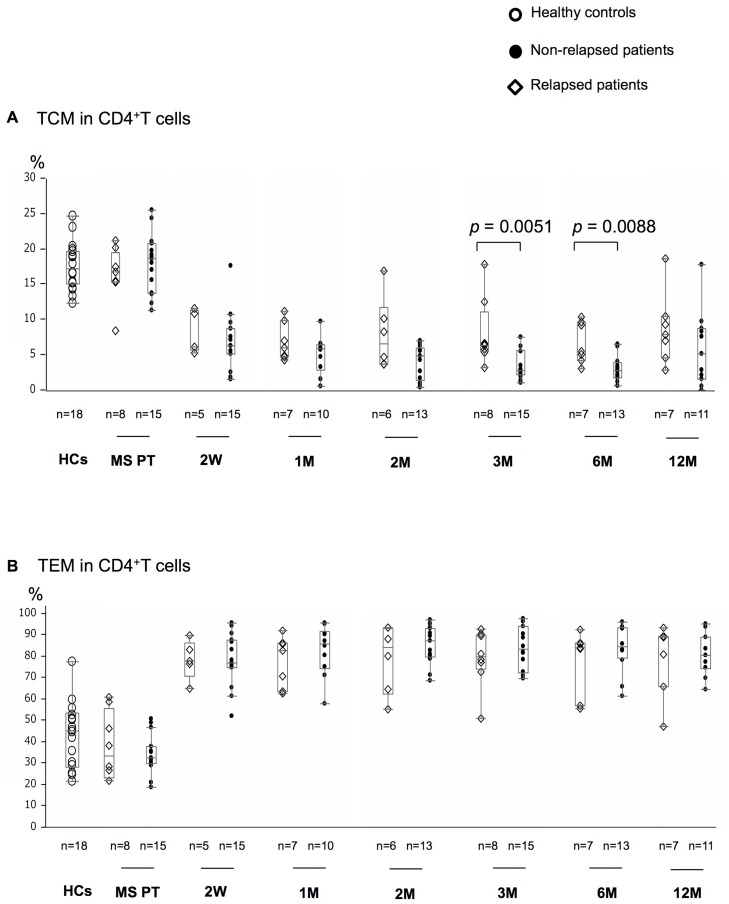
The relapsed patients have greater percentages of CD4^+^TCM at 3 and 6 months. Comparisons of TCM (A) and TEM (B) percentages in CD4^+^T cells at pre-treatment (MS PT) and the indicated periods of fingolimod treatment between MS patients with (open diamonds) and without (closed circles) relapse during the therapy. Box-whisker plots are shown. W = week; M = month.

When the relapsed and non-relapsed MS patients were compared, only the percentages of CD4^+^TCM differed significantly between the two groups. Specifically, the relapsed patients had significantly greater percentages of CD4^+^TCM than the non-relapsed patients at 3 and 6 months (*p* = 0.0051 and *p* = 0.0088, respectively) (Fig 4).

## Discussion

This study is the first to successively assess the dynamics of T cell subsets from 2 weeks up to 12 months of fingolimod therapy in MS patients. Although selection bias was not completely eliminated, no selection was made on referral to our clinic for initiation of fingolimod treatment. In addition, clinical and MRI relapses were seen in 8.7% and 26.1% of our patients, respectively, at 0–12 months. These findings are consistent with the observations in a Japanese clinical trial of fingolimod 0.5 mg once daily, in which clinical relapses occurred in 15.6% at 0–6 months and 6.7% at 7–12 months, while gadolinium-enhanced T1 lesions appeared in 22.2% at 0–6 months and 15.6% at 7–12 months and new/enlarging T2 lesions appeared in 33.3% at 0–6 months and 13.3% at 7–12 months [20]. Therefore, we believe that the recruitment of MS patients did not severely distort our findings.

The reductions in TCM and naïve T cells in both CD4^+^T and CD8^+^T cells in peripheral blood occurred very rapidly and continued for as long as fingolimod was administered. Interestingly, among the T cell subsets showing significant alterations during fingolimod therapy, only the CD4^+^TCM percentages were associated with MS relapse, although the pre-treatment TCM percentages in CD4^+^T cells did not differ significantly between the relapsed and non-relapsed patients, suggesting a critical role of CD4^+^TCM cells in MS relapse. The relapsed patients had a shorter disease duration and higher Progression Index than the non-relapsed patients in our study, but the differences were not significant, possibly because of the small sample size. Thus, MS patients in the early course, who are presumed to have high inflammatory disease activity, may tend to show a suboptimal response to fingolimod 0.5 mg once daily because of an insufficient decrease in CD4^+^TCM cells. Therefore, careful observation of the clinical and MRI disease activity may be required when fingolimod is introduced in MS patients with shorter disease duration and high disease activity. The present findings indicate that peripheral blood CD4^+^TCM cell percentages during fingolimod therapy may have a predictive value for relapse at least during the first year. However, as the present study involved a relatively small cohort, a large-scale confirmatory study will be necessary to establish TCM frequency as a potential biomarker for the relapse propensity of MS patients on fingolimod treatment.

The increased pre-treatment percentages of IL17-producing CD4^+^T and CD8^+^T cells in MS patients, especially in MS patients without IFNβ or corticosteroids, are consistent with a previous study showing that peripheral blood Th17 cells increase in Western MS patients [21]. These observations collectively suggest pathogenic roles of IL17-producing T cells in MS. In our study, the IL17-producing CD4^+^T cell percentage showed a transient increase in the very early course of fingolimod therapy, which is partly compatible with the increase in IL17-producing cells observed in a short-term study of fingolimod [9]. This increase may be explained by a relative increase in TEM, which contain both Th1 and Th17 cells [10], while the absence of replenishment of Th1 and Th17 cells by naïve T cells that are continuously trapped in lymph nodes may limit the persistence of the increase [6,22]. Importantly, this transient increase in the IL17-producing cell percentage was not associated with the development of relapse, at least in anti-AQP4 antibody-seronegative MS patients. In contrast, unexpected relapse was reported to occur in the early course of anti-AQP4 antibody-positive patients with neuromyelitis optica (NMO) [19,23] and tumefactive MS patients [24,25]. As an increase in IL17 in CSF was observed in NMO patients at relapse [26–28], it is possible that the transient increase in the IL17-producing cell percentage may play a role in these conditions with fingolimod therapy.

Compared with HCs, IFNγ-, IL4-, and IL9-producing CD4^+^T and CD8^+^T cells were increased in MS patients at pre-treatment, especially in the MS patients without IFNγ or corticosteroids. Increases in IFNγ-producing CD4^+^T and CD8^+^T cells were previously reported in Western and Asian MS patients [29,30], consistent with the induction of MS relapse by administration of IFNγ [31]. The increase in IL4-producing cells in the remission phase of MS patients may be a host defence mechanism. In our hands, fingolimod did not appear to down-regulate IL4-producing cells, which may be beneficial for Th1/Th17-mediated diseases like MS [1]. IL9-producing cells at pre-treatment may have been influenced by disease-modifying therapies such as IFNβ [32], which was reported to potentiate IL9 production via IL21 in Th9-polarising conditions in humans [33]. However, as the MS patients without IFNβ also had greater percentages of IL9-producing CD4^+^T and CD8^+^T cells, such increases may have some relevance to MS mechanisms. Although IL9 was previously regarded as one of the Th2 type cytokines, it was recently reported that IL9-producing CD4^+^T cells can induce neuroinflammation in certain conditions [34] and that Th17 cells can produce IL9, which exacerbates experimental autoimmune encephalomyelitis [35]. Therefore, increases in IL9-producing T cells in MS patients may warrant to careful monitoring to clarify the relationship between IL9-producing T cell changes and relapse while on fingolimod therapy.

Finally, Treg and Ts are subpopulations of T cells that down-regulate immune response and autoimmune disease activities [36,37]. However, we did not find any differences in these cell percentages between the relapsed and non-relapsed MS patients during fingolimod therapy. Although the relative increases in these cells may have some roles in down-modulating the disease activity of MS during fingolimod treatment, these cell percentages are not useful as predictors of relapse.

## Supporting Information

S1 Dataset Actual values for percentages and counts of T cell subsets in healthy controls and patients with multiple sclerosis. (Sheet 1) The rows show the actual values for the percentages and counts of T cell subsets in individual samples. T cells are classified by their surface antigens. H1–H18 are healthy controls (HCs) and P1–P23 are patients with multiple sclerosis (MS). Figs 1, 2, 4, and S2 are based on these values. (Sheet 2) The rows show the actual values for the percentages and counts of T cell subsets in individual samples. T cells are classified by their cytokine productions. H1–H9 are HCs and P1–P16 are patients with MS. Figs 3, S3, and S4 are based on these values. The clinical statuses and timings of sample collection before or after induction of fingolimod treatment are also shown in each patient. EDSS = expanded disability status scale of Kurtzke; F = female; M = male; NT = not treated; WBC = white blood cells.
(XLSX)

S1 Fig Gating of cells in the flow cytometry studies. First, lymphocytes were gated as forward scatter-medium and side scatter-low populations (A), and CD4^+^T cells were gated as indicated in (B). The cells were then gated to discriminate central memory T cells (TCM), effector memory T cells (TEM), regulatory T cells (Treg), and suppressor precursor T cells (Ts). To determine the threshold for the CCR7^+^ and CCR7^-^ populations, we used a PE-conjugated mouse IgG1 isotype control (C). The cells were then separated by CD45RO and CCR7 to detect CCR7^+^CD45RO^+^ TCM, CCR7^-^CD45RO^+^ TEM, and CCR7^+^CD45RO^-^ naïve T cells (D). To determine the threshold for the CD25^+^ and CD25^-^ populations, we used a PE-conjugated mouse IgG2b isotype control (E). The CD4^+^ populations were then separated by CD25 and CD127 to discriminate CD127^low^CD25^high^ Treg (F). We also used an APC-conjugated mouse IgG1 isotype control (G) to determine the threshold for the CD28^+^ and CD28^-^ populations to separate CD4^+^CD28^-^ Ts (H).
(TIFF)

S2 Fig The counts of T cell subsets are significantly decreased from 2 weeks. Effects of fingolimod on the absolute counts of phenotypically distinct CD4^+^T (A) and CD8^+^T (B) cell subpopulations in healthy controls (HCs) and MS patients at pre-treatment (MS PT) and the indicated periods of fingolimod treatment. Naïve = naïve T cells (CCR7^+^CD45RO^-^); TCM = central memory T cells (CCR7^+^CD45RO^+^); TEM = effector memory T cells (CCR7^-^CD45RA^-^); Treg = regulatory T cells (CD4^+^CD25^high^CD127^low^); Ts = suppressor precursor T cells (CD28^-^); TEMRA = CD8^+^CD45RA^+^ effector memory T cells (CD8^+^CCR7^-^CD45RA^+^). The numbers analysed were: HC = 18, and MS PT = 23, 2W = 20, 1M = 17, 2M = 19, 3M = 23, 6M = 20, 12M = 18. The horizontal bars indicate the mean values. W = week; M = month. ***p<0.0001, **p<0.01, *p<0.05.
(TIFF)

S3 Fig IFNγ-producing cells at pre-treatment are only significant in the MS patients without IFNβ or corticosteroids. Percentages of IFNγ- (A), IL17- (B), IL9- (C), and IL4-producing (D) cells among CD4^+^T and CD8^+^T cells in MS patients with (MS PT+) and without (MS PT-) IFNβ or corticosteroids within 3 months of the initiation of fingolimod. The numbers examined at each time point were: HC = 8, and MS PT- = 6, MS PT+ = 10. Box-whisker plots are shown.
(TIFF)

S4 Fig The absolute counts of all cytokine-producing T cells in CD4^+^T cells are decreased from 2 weeks. Effects of fingolimod on the absolute counts of IFNγ- (A), IL17- (B), IL9- (C), and IL4-producing (D) cells in CD4^+^T and CD8^+^T cells in healthy controls (HCs) and MS patients at pre-treatment (MS PT) and the indicated periods of fingolimod treatment. The numbers examined at each time point were: HC = 9, and MS PT = 16, 2W = 12, 1M = 12, 2M = 11, 3M = 12, 6M = 7, 12M = 11. The horizontal bars indicate the mean values. W = week; M = month. **p<0.01, *p<0.05.
(TIFF)
