# Supplementary figures and images for: Peripheral Blood T Cell Dynamics Predict Relapse in Multiple Sclerosis Patients on Fingolimod
Source: PLoS One. 2015 Apr 28;10(4):e0124923. doi: 10.1371/journal.pone.0124923 (PMC4412716; doi:10.1371/journal.pone.0124923)

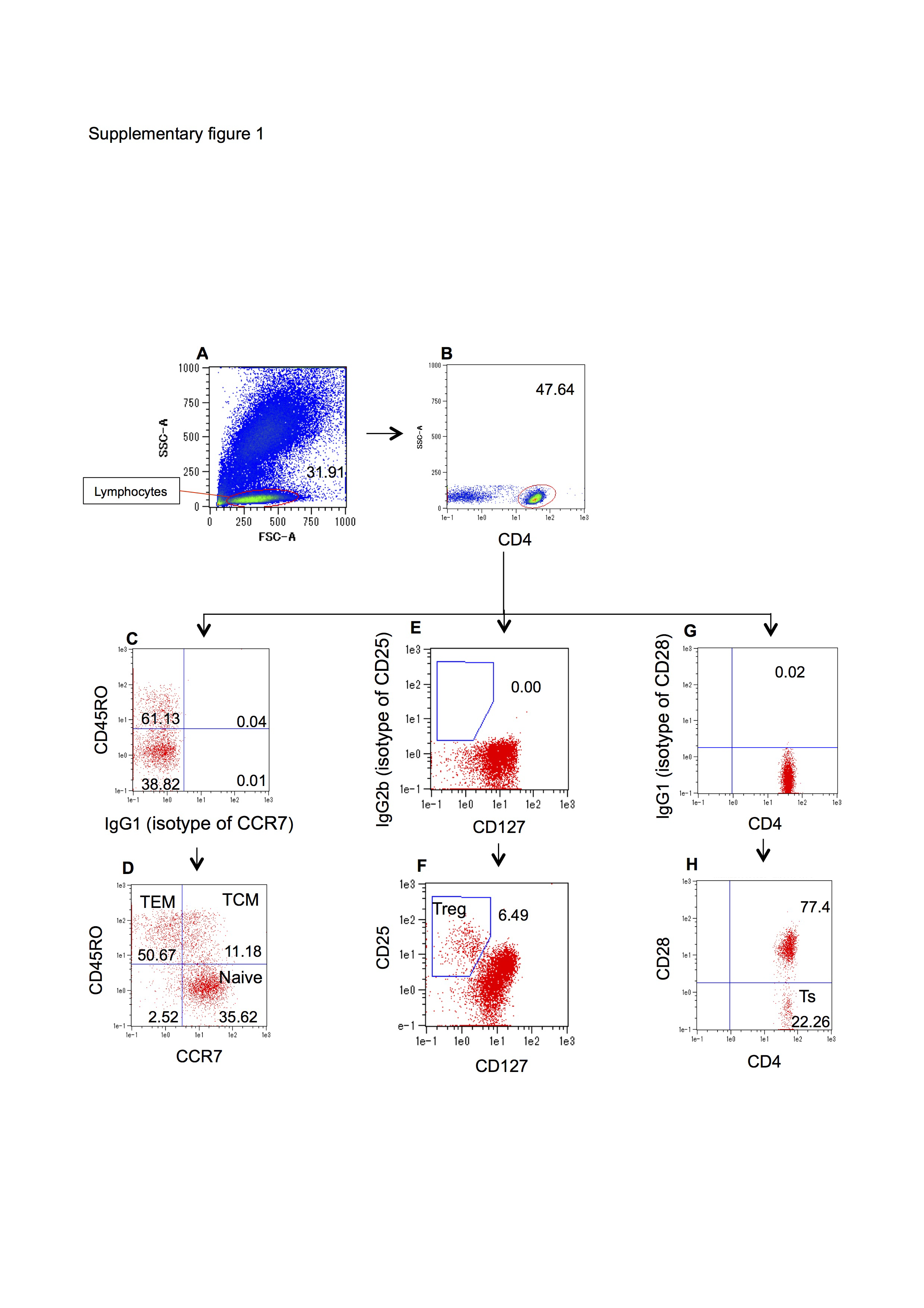

Supplement: S1 Fig — First, lymphocytes were gated as forward scatter-medium and side scatter-low populations (A), and CD4+T cells were gated as indicated in (B). The cells were then gated to discriminate central memory T cells (TCM), effector memory T cells (TEM), regulatory T cells (Treg), and suppressor precursor T cells (Ts). To determine the threshold for the CCR7+ and CCR7- populations, we used a PE-conjugated mouse IgG1 isotype control (C). The cells were then separated by CD45RO and CCR7 to detect CCR7+CD45RO+ TCM, CCR7-CD45RO+ TEM, and CCR7+CD45RO- naïve T cells (D). To determine the threshold for the CD25+ and CD25- populations, we used a PE-conjugated mouse IgG2b isotype control (E). The CD4+ populations were then separated by CD25 and CD127 to discriminate CD127lowCD25high Treg (F). We also used an APC-conjugated mouse IgG1 isotype control (G) to determine the threshold for the CD28+ and CD28- populations to separate CD4+CD28- Ts (H). (TIFF) [file pone.0124923.s002.tiff]

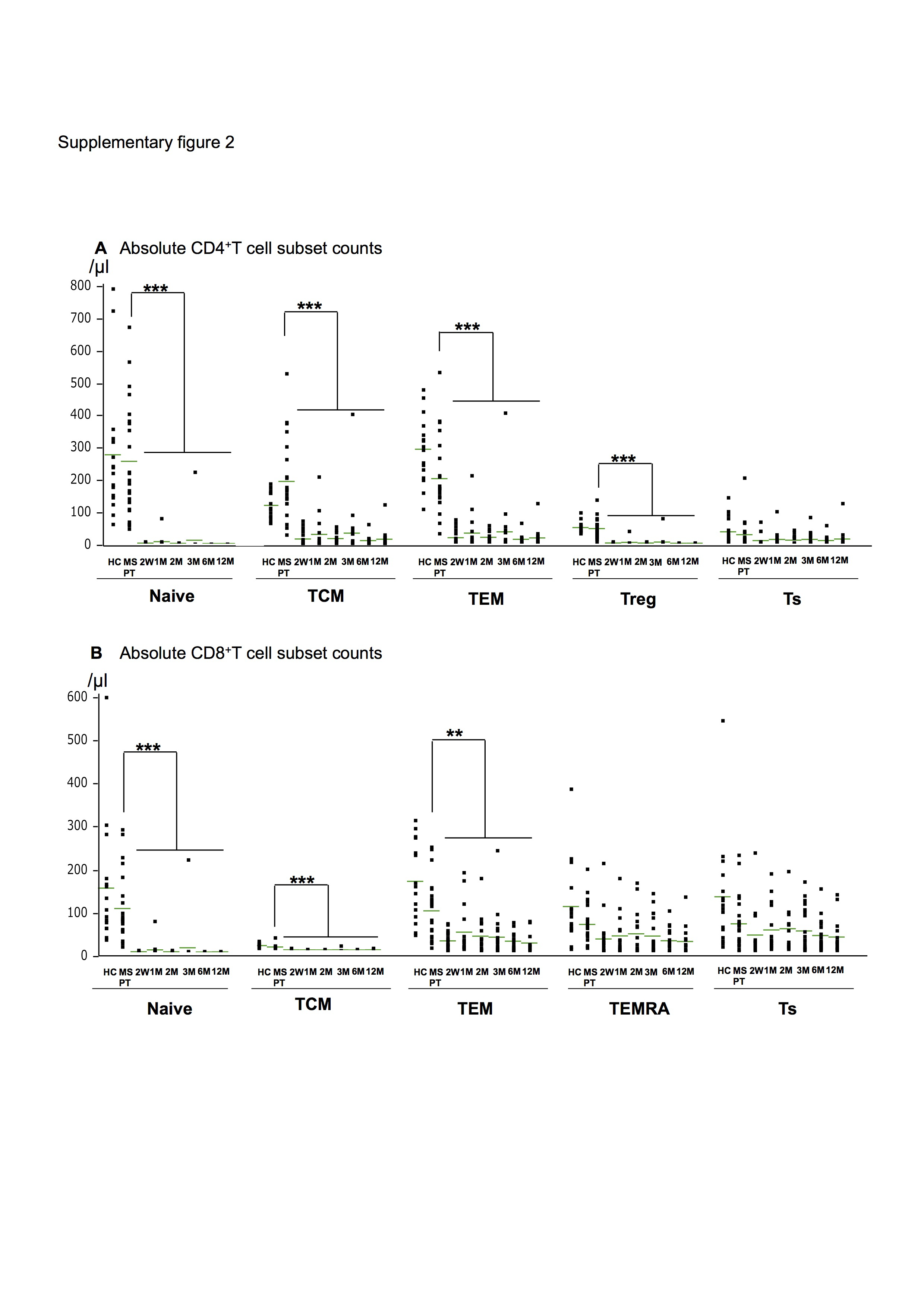

Supplement: S2 Fig — Effects of fingolimod on the absolute counts of phenotypically distinct CD4+T (A) and CD8+T (B) cell subpopulations in healthy controls (HCs) and MS patients at pre-treatment (MS PT) and the indicated periods of fingolimod treatment. Naïve = naïve T cells (CCR7+CD45RO-); TCM = central memory T cells (CCR7+CD45RO+); TEM = effector memory T cells (CCR7-CD45RA-); Treg = regulatory T cells (CD4+CD25highCD127low); Ts = suppressor precursor T cells (CD28-); TEMRA = CD8+CD45RA+ effector memory T cells (CD8+CCR7-CD45RA+). The numbers analysed were: HC = 18, and MS PT = 23, 2W = 20, 1M = 17, 2M = 19, 3M = 23, 6M = 20, 12M = 18. The horizontal bars indicate the mean values. W = week; M = month. ***p<0.0001, **p<0.01, *p<0.05. (TIFF) [file pone.0124923.s003.tiff]

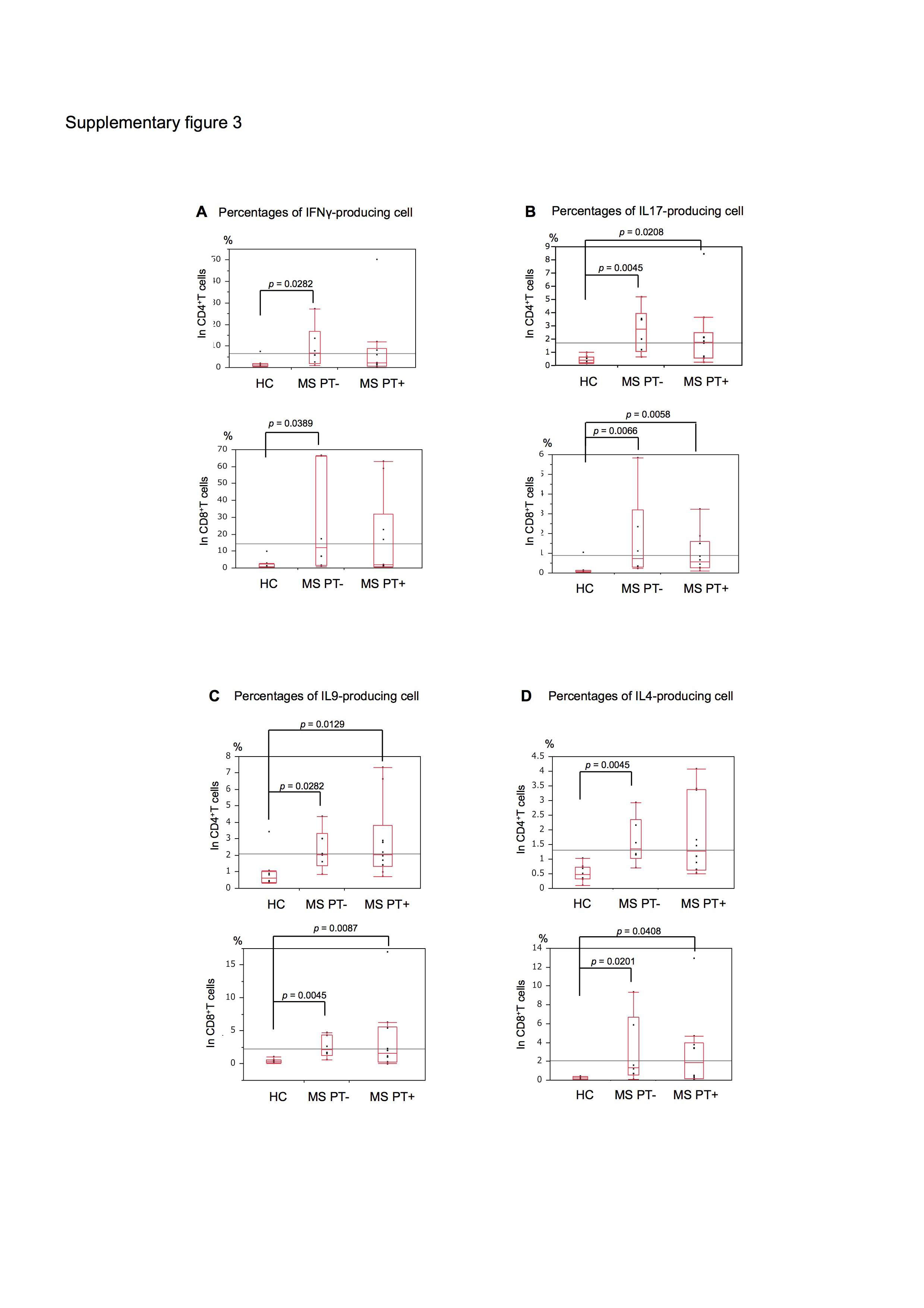

Supplement: S3 Fig — Percentages of IFNγ- (A), IL17- (B), IL9- (C), and IL4-producing (D) cells among CD4+T and CD8+T cells in MS patients with (MS PT+) and without (MS PT-) IFNβ or corticosteroids within 3 months of the initiation of fingolimod. The numbers examined at each time point were: HC = 8, and MS PT- = 6, MS PT+ = 10. Box-whisker plots are shown. (TIFF) [file pone.0124923.s004.tiff]

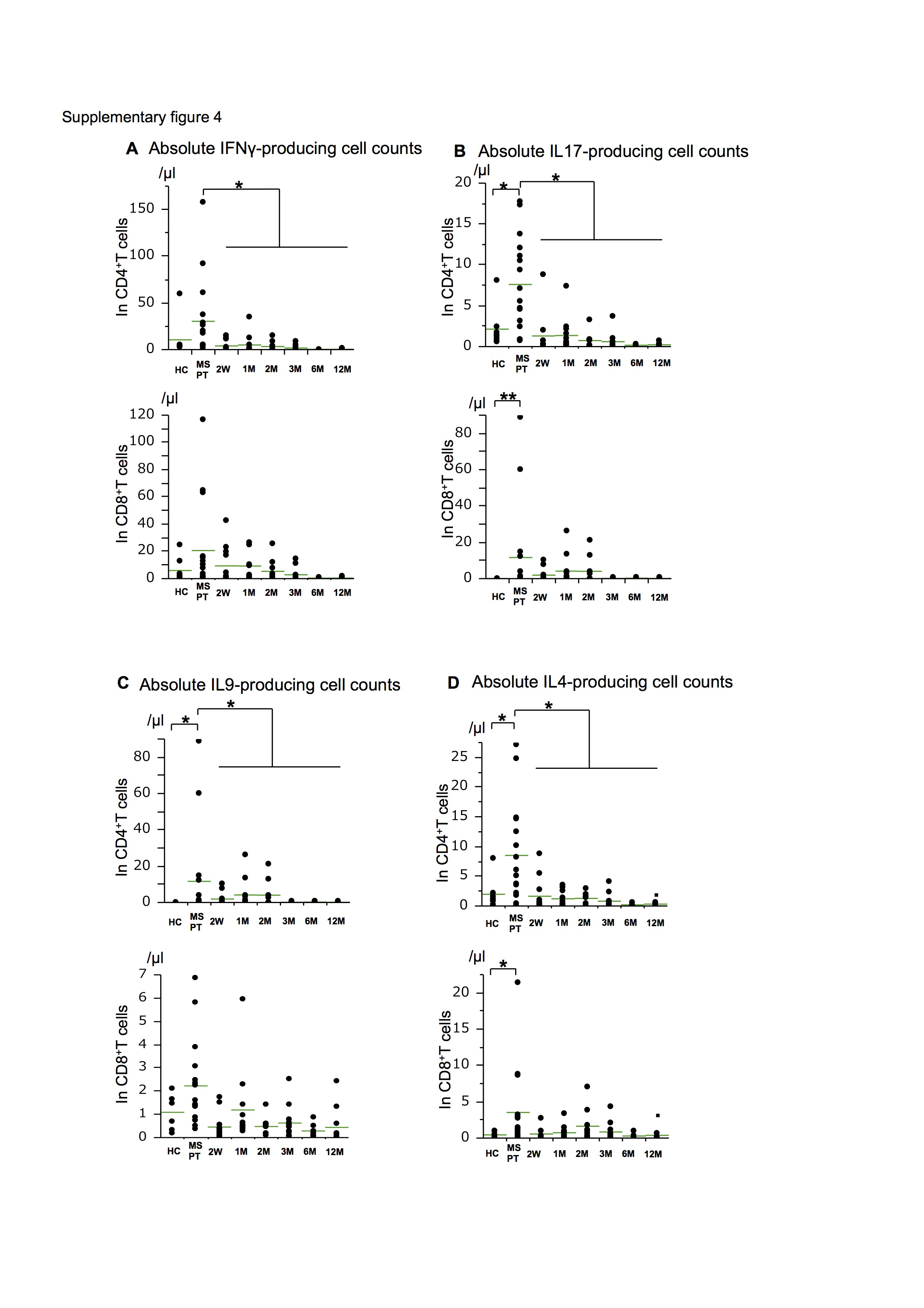

Supplement: S4 Fig — Effects of fingolimod on the absolute counts of IFNγ- (A), IL17- (B), IL9- (C), and IL4-producing (D) cells in CD4+T and CD8+T cells in healthy controls (HCs) and MS patients at pre-treatment (MS PT) and the indicated periods of fingolimod treatment. The numbers examined at each time point were: HC = 9, and MS PT = 16, 2W = 12, 1M = 12, 2M = 11, 3M = 12, 6M = 7, 12M = 11. The horizontal bars indicate the mean values. W = week; M = month. **p<0.01, *p<0.05. (TIFF) [file pone.0124923.s005.tiff]
